# RECQ1 Helicase in Genomic Stability and Cancer

**DOI:** 10.3390/genes11060622

**Published:** 2020-06-05

**Authors:** Subrata Debnath, Sudha Sharma

**Affiliations:** 1 Department of Biochemistry and Molecular Biology, College of Medicine, Howard University, 520 W Street, NW, Washington, DC 20059, USA; subrata.debnath@howard.edu; 2 National Human Genome Center, College of Medicine, Howard University, 520 W Street, NW, Washington, DC 20059, USA

**Keywords:** helicase, replication, DNA repair, G4, transcription, genomic stability, breast cancer, cancer

## Abstract

RECQ1 (also known as RECQL or RECQL1) belongs to the RecQ family of DNA helicases, members of which are linked with rare genetic diseases of cancer predisposition in humans. RECQ1 is implicated in several cellular processes, including DNA repair, cell cycle and growth, telomere maintenance, and transcription. Earlier studies have demonstrated a unique requirement of RECQ1 in ensuring chromosomal stability and suggested its potential involvement in tumorigenesis. Recent reports have suggested that *RECQ1* is a potential breast cancer susceptibility gene, and missense mutations in this gene contribute to familial breast cancer development. Here, we provide a framework for understanding how the genetic or functional loss of RECQ1 might contribute to genomic instability and cancer.

## 1. Introduction

Mutations in DNA repair genes elicit genome instability, leading to cellular transformation and the development of cancer [1]. This class of genes may also include RecQ helicases, represented by five distinct homologs in humans, because of their caretaker function in genome maintenance [2]. Indeed, loss of function mutations in genes encoding RecQ helicases BLM, WRN, and RECQL4 are causatively linked with rare genetic syndromes and cancer predisposition [3,4]. Despite being the first discovered RecQ homolog in humans and essential for chromosomal stability, the biological significance of RECQ1 (also known as RECQL or RECQL1) in human health and disease has remained underappreciated. Recent discoveries suggest that rare, recurrent germline mutations in *RECQ1* significantly increase the risk of breast cancer [5,6]. Here, we summarize the findings on the RECQ1 helicase to gain a better understanding of its functions in genomic stability maintenance that could be critical in tumorigenesis.

## 2. Structure and Biochemical Properties of RECQ1

**Figure 1:**
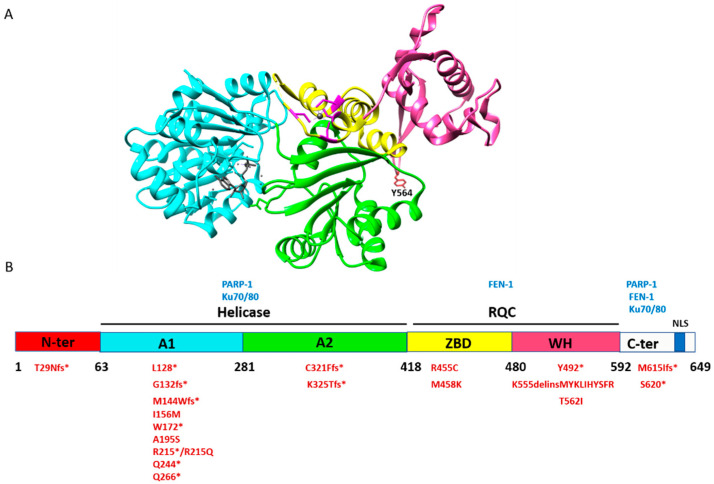
Human RECQ1 protein. (**A**) Crystal structure of RECQ1 (49–616) in the presence of MgCl_2_, ATPγS, and DNA (PDB: 2V1X). A1 (amino acids 63–281; cyan color), A2 (amino acids 282–418; green color), ZBD (amino acids 418–480; yellow color), and WH (amino acids 481–592; pink color). The conserved cysteine residues C453, C471, C475, and C478, shown by magenta color, are coordinated with Zinc ion (dim gray). The Y564 in β hairpin is shown by red color. The crystal structure contains Mg-ADP, but the DNA is missing. (**B**) Schematic of the RECQ1 domain structure. Breast cancer risk associated variants and the RECQ1 domains known to interact with various proteins are indicated. Protein interaction between RECQ1 and PARP-1 is mediated primarily through the helicase domain and C-terminus, FEN-1 interacts with the RQC domain and C-terminus, and the interaction with Ku70/80 is mediated via the RECQ1 C-terminus, with some contribution from the helicase domain. NLS—nuclear localization sequence.

The human *RECQ1* gene, located on chromosome 12p12, encodes a 649 amino acid protein of 73 kDa [7,8]. RECQ1 protein is expressed ubiquitously and represents the most abundant RecQ homolog present in humans [9]. It contains four domains: N-terminus (amino acid residues 1–62), a highly conserved core helicase domain (amino acid residues 63–418), the RecQ-specific C-terminal (RQC) domain (amino acid residues 419–592), and C-terminus (amino acid residues 593–694) [3] (Figure 1). The Helicase and RNaseD C-terminal (HRDC) domain, which is present in several RecQ proteins like bacterial RecQ and human WRN and BLM, is absent in RECQ1 [10]. Biochemically, RECQ1 catalyzes ATP-dependent unwinding of DNA, with a 3′-5′ polarity, and can unwind a variety of DNA structures other than B-form DNA duplexes, such as forked duplexes, D-loops, and Holiday Junction (HJ) structures [11]. In addition to helicase activity, RECQ1 also promotes annealing of complementary single-strand DNA in an ATP-independent manner [11,12]. These seemingly opposite dual activities are guided by ATP binding and the oligomeric states of human RECQ1 [13]. RECQ1 protein forms two quaternary structures: the higher-order oligomers consistent with hexamers and pentamers are associated with single-strand DNA annealing, whereas lower-order oligomers, consistent with dimers and monomers, are associated with duplex DNA unwinding [13]. The observation that the truncated RECQ1 (amino acid residues 49–616) lacks a single-strand annealing property indicates that the oligomerization signals for RECQ1 are in the N-terminal region [14,15,16].

The crystal structure of RECQ1 (amino acid residues 49–616) [14], in the presence of MgCl_2_, ATPγS, and DNA (PBD ID: 2V1X), exhibits four structurally defined domains. The helicase domain of RECQ1 consists of two Rec-A like domains, A1 (amino acid residues 63–281) and A2 (amino acid residues 282–418), containing the highly conserved signature helicase motifs of SF-2 superfamily and the nucleotide-binding pocket. The RQC domain of RECQ1 is defined by two separately folded domains, namely, Zn-binding domain (ZBD; amino acid residues 419–480) and Winged helix (WH) domain (amino acid residues 481–592). The ZBD consists of highly conserved 4 cysteine zinc-binding motif and two anti-parallel α-helices (HH) [14] (Figure 1A). Alteration of these cysteine residues changes the overall conformation of RECQ1 protein and impairs its helicase and ATPase activities, whereas the strand-annealing activity is minimally affected [15,16]. The WH domain of RECQ1 has a β-hairpin loop and a tyrosine residue (Y564) at the tip of the hairpin, which is important for the DNA unwinding and ATPase activity, indicating the importance of this region and the residue in the enzyme’s helicase function [14]. However, these mutants retain the strand annealing activity of RECQ1 [15].

## 3. Demonstrated Roles of RECQ1 in DNA Repair

RECQ1-deficiency in mice [17] and human cells [18] causes constitutive chromosomal instability, sister chromatid exchanges, increased DNA double-strand breaks (DSBs), and accumulation of unresolved recombination intermediates, suggesting a role in DNA damage response and repair. RECQ1-deficient cells accumulate DNA damage and display increased sensitivity to DNA damaging agents that induce stalled and collapsed replication forks, oxidative damage, and DSBs [18,19,20,21,22]. The data so far suggest that RECQ1 employs its multiple catalytic actions and interactions with specific protein partners in ensuring genome stability [9]. The first identified interaction of RECQ1 was with the nuclear import protein importin-α homologs Qip1 and Rch1 [23]. A list of known RECQ1 protein partners is shown in Table 1.

We highlight below some of the well-characterized physical and functional interactions of RECQ1, mediating its various roles in DNA repair and genome maintenance.

### 3.1. RECQ1–RPA Interaction

Replication protein A (RPA) is a single-strand DNA binding protein and a key player of cellular nucleic acid metabolism [32]. Notably, the interaction of RecQ helicases with the heterotrimeric RPA is conserved [3]. RPA interacts with RECQ1 and stimulates its DNA unwinding activities [30] while inhibiting its annealing activities [11]. The RECQ1 helicase activity stimulated by RPA is biochemically important since RECQ1 is not a very processive helicase and fails to unwind duplex-DNA longer than 100 bp. However, in the presence of RPA, it unwinds much longer duplex-DNA, such as a 500 bp [30]. The Brosh lab reported the role of RECQ1 in maintaining free RPA availability to coat nascent single-strand DNA during replication stress and, therefore, in the repair of replication-associated DSBs [33]. When replication forks stall, for example, under gemcitabine treatment, RECQ1 helicase aids in RPA accumulation by revealing tracts of single-strand DNA at stalled forks, resulting in checkpoint activation, DNA damage repair, and cell survival [34]. Thus, functional interaction with RPA is important for RECQ1 functions in DNA replication, repair, and recombination.

### 3.2. RECQ1–PARP-1 Interaction

Poly [ADP-ribose] polymerase 1 (PARP-1) is the first responder that detects DNA damage and it is also involved in the regulation of several DNA repair processes. Endogenous RECQ1 exists in a common complex with PARP-1 and RPA [22]. The direct physical interaction of PARP-1 protein with RECQ1 is mediated primarily through its C-terminus and the helicase domain [22]. Elegant work from the Vindigni lab demonstrated that PARP-1 is critical in regulating ATPase and branch migration activities of RECQ1 for the restart of regressed replication forks, following Topoisomerase 1(TOP1) inhibition by camptothecin [19]. Our recent work shows that RECQ1 helicase activity is important for cancer cell survival after camptothecin-induced DNA damage [34]. These discoveries suggest that RECQ1 may be a new therapeutic target and could modulate the efficacy of combinational cancer therapies with PARP-1 and TOP1 inhibitors. Recently, we discovered a new subpathway of conventional long-patch base excision repair (LP-BER) that is mediated by RECQ1 and ERCC1-XPF endonuclease in cooperation with PARP-1 and RPA to repair base damage such as oxidation and alkylation [29]. We have shown that RECQ1 inhibits the auto-ADP-ribosylation of PARP-1, which inhibits single-nucleotide (SN)-BER and promotes RECQ1-dependent LP-BER as the preferred mechanism for repair [29]. PARP-1 and RECQ1 also interact with the common components of the mismatch repair system [26,35], suggesting a possible role in the suppression of homologous recombination (HR). Thus, the RECQ1–PARP-1 partnership is critical for both replication restart and DNA repair.

### 3.3. RECQ1–Ku70/80 Interaction

Human Ku is a heterodimeric protein composed of two subunits Ku70 and Ku80 that, in complex with DNA-dependent protein kinase catalytic subunit (DNA–PKcs), constitutes the full DNA-dependent protein kinase (DNA-PK) [36]. Ku binds to the DNA ends and functions as a molecular scaffold to which other proteins involved in nonhomologous end joining (NHEJ) can bind and repair the DSB [36]. RECQ1 directly interacts with the Ku70/Ku80 subunit of the DNA–PK complex [28]. RECQ1 regulates the binding of Ku70/80 to the DNA in vitro and modulates the NHEJ pathway of DSB repair [28]. Unpublished results from our lab suggest physical interaction of RECQ1 with the MRE11–RAD50–NBS1 (MRN) complex, further strengthening its role in the repair of DSBs.

### 3.4. RECQ1–FEN-1 Interaction

Flap Endonuclease-1 (FEN-1) is a structure-specific nuclease that cleaves 5′ flaps of the branched DNA structures and possesses double-strand specific 5′-3′ exonuclease activity [37]. FEN-1 is involved in base excision repair (BER) by removing 5′ flap structures formed during gap-filling DNA synthesis and in the processing of the 5′ ends of Okazaki fragments during lagging strand DNA synthesis [37]. Enzymatic functions of FEN-1 are critical for telomeric lagging strand DNA synthesis and telomere stability [38]. We have shown that like WRN and BLM [39], RECQ1 also interacts with FEN-1 and stimulates its 5′-flap endonuclease activity. FEN-1 interacts with RECQ1 primarily through its RQC domain and C-terminus in a DNA-independent manner [27]. Furthermore, RECQ1 binds to telomeres and facilitates the constitutive binding of FEN-1 to telomers in replicating cells [27]. Consistent with a role of RECQ1 in telomere stability, the Bohr lab reported breakage and loss of telomeres in RECQ1-depleted cells [40].

## 4. Non-Canonical Roles of RECQ1

In addition to its established role in DNA repair, increasing evidence points towards a function of RECQ1 in the regulation of gene expression [41,42]. RecQ family members WRN, BLM, and RECQL5 have been shown to modulate gene expression [43,44,45,46]. Our work has shown that genes downregulated upon RECQ1 silencing displayed an enrichment of predicted G4-forming sequences in their promoter elements [41]. Although the mechanism of RECQ1-regulated gene expression is not fully understood, binding to the G4 motifs in gene promoters was suggested [41]. How could RECQ1 binding to the G4 motifs in the promoters of its target genes result in transcription control? Unlike WRN and BLM helicases, RECQ1 is poorly active as a helicase on G4 DNA substrates [47]. Thus, the functional significance of RECQ1′s interaction with promoter-associated G4 DNA is unclear. Coregulation of gene expression by RECQ1 and the sequence-related WRN and BLM helicases suggest that potentially overlapping roles of human RecQ helicases in the regulation of gene expression [3] may contribute to the complexity of the DNA damage response. The guanine residues in GC-rich promoter sequences that adopt G4 structure are susceptible to oxidation. The 8-oxoG in the G4 motif in gene regulatory regions has been shown to couple DNA repair with transcriptional regulation [48,49,50]. A potential function of RECQ1 at G4 motifs could be to recognize and repair oxidative lesions, 8-oxoG, in the G4 motif. Considering that transcriptional response to oxidative challenge is prompt and our data showing rapid recruitment of RECQ1 to oxidized chromatin [22], we hypothesize that RECQ1 helicase is utilized in transcriptional gene activation in response to 8-oxoG lesions. RECQ1 may also regulate gene expression as a component of the constitutive replication stress response in tumor cells that undergo rapid proliferation.

Thus, the proposed roles of RECQ1 in DNA repair and gene regulation may not be mutually exclusive. RECQ1 may be involved in transcription regulation either by direct binding to DNA, alteration of chromatin, or through yet-uncharacterized interaction with specific transcription factors. In this regard, RECQ1 was identified in chromatin-associated and soluble complexes of the members of Forkhead Box family of transcription factors [51] as well as in complex with the chromatin regulator protein barrier to autointegration factor (BAF) [24], and with DNA damage-binding protein 1 (DDB1) for transcription directed by HomoID box, a core promoter element [25]. The basic composition of the RECQ1 complex(es) that exist in a given cell type or under given physiological conditions is yet unknown, but it is conceivable that RECQ1 is present in several distinct complexes, each of which may have a unique function. Further work is needed to investigate the mechanism of RECQ1-dependent gene regulation and its relevance to genome maintenance.

## 5. Association of RECQ1 Expression with Cancer

RECQ1 is overexpressed in transformed cells and a variety of cancers [52]. Cancer cell-specific upregulation of RECQ1 has been demonstrated in glioblastoma [53], multiple myeloma [54], ovarian cancers [55], hematological cancers [56], hepatocellular carcinoma [57], head and neck [58], and tongue squamous cell carcinoma [59]. Silencing RECQ1 in cancer cells induces cell death by mitotic catastrophe [60] and reduced tumor growth in mouse models [61]. Furthermore, using zebrafish as a model organism, a recent study demonstrated that silencing RECQ1 reduces the tumor growth rate of glioblastoma cells, U87 [62]. Transient depletion of RECQ1 using siRNA decreases cancer cell migration and invasion in breast cancer MDA-MB-231 cells by regulating the expression of genes associated with cancer progression [41,42]. Moreover, silencing RECQ1 in squamous cell carcinoma reduced the expression of immunosuppressive factors, namely, IL-10 and VEGF, that regulate cancer cell migration [59]. Depletion of RECQ1 sensitizes cancer cells to a variety of chemotherapeutics, namely, camptothecin [19], psoralen, hydroxyurea [20], temozolomide [29], melphalan [54] and gemcitabine [34], suggesting that RECQ1′s functions in DNA repair are important in cellular resistance to these therapeutic agents in cancer cells. In The Cancer Genome Atlas (TCGA) dataset for invasive breast carcinomas, RECQ1 expression is altered mostly by mRNA upregulation and copy number amplification in about 10% of patients. In a large METABRIC cohort of sporadic breast cancer (*n* = 1977), we found that altered RECQ1 expression is associated with aggressive breast cancers and poor prognosis. In this cohort, high RECQ1 is associated with poor survival in patients with estrogen receptor-negative tumors that received anthracycline-based chemotherapy [63]. Moreover, RECQ1 expression is upregulated in response to DNA damage, including those induced by alkylating and other chemotherapeutic agents [64]. Given that the RECQ1 protein restarts replication forks stalled by DNA damaging agents and allows some cancer cells to survive the cytotoxic effect of this class of chemotherapeutic drugs, the downregulation of RECQ1 could be effective in treating cancer patients.

These results collectively suggest that RECQ1 functions, as well as its expression levels, are critical in cancer pathogenesis and the patient’s response to anticancer therapy. Therefore, it is important to elucidate the absolute functions of RECQ1 in DNA repair and tumor suppression and understand how defects in the biological functions of RECQ1 protein promote cancer risk.

## 6. *RECQ1* as a Candidate Breast Cancer Susceptibility Gene

In 2015, two independent studies linked mutations in *RECQ1* to increased breast cancer risk [5,6]. Cybulski et al. used exome sequencing to discover that germline mutations in *RECQ1* increase an individual’s risk of developing hereditary breast cancer by 5-fold among cases from Poland and by 16-fold in a Quebec population. They discovered two previously unknown germline truncating mutations; one only in Polish individuals, and the other only in French-Canadian individuals. In Quebec, 7 of 1013 patients with high-risk breast cancer carried the c.643C>T (p.R215*) variant compared with 1 of 7136 newborns in Quebec (odds ratio (OR) = 49.3, *p* < 10^−5^, two-tailed Fisher’s exact test). In Poland, 30 of 13,136 unselected breast cancer cases carried the c.1667_1667+3delAGTA (p.K555delinsMYKLIHYSFR) variant, compared with 2 of 4702 controls (OR = 5.4, *p* = 0.008, two-tailed Fisher’s exact test) [5]. This variant deleted K555 and added 10 amino acids that displaced a conserved β-hairpin, which is essential for RECQ1 helicase activity [14]. In the same year, Sun et al., in an independent study from China, found nine potentially pathogenic mutations: three nonsense mutations leading to a truncated protein (p.L128*, p.W172*, and Q266*), one splicing mutation (c.395-2A>G), and five deleterious missense mutations disrupting helicase activity (p.A195S, p.R215Q, p.R455C, p.M458K, and p.T562I) among 448 patients who had tested negative for *BRCA1* and *BRCA2* mutations, compared with 1 of 1588 controls (p.R455C; OR = 31.9, *p* < 10^−5^, two-tailed Fisher’s exact test) [6].

After these two initial reports, Kong et al., in 2016, studied 1110 breast cancer patients from Hong Kong who had previously tested negative for *BRCA1*, *BRCA2*, *TP53,* and *PTEN* mutations. They found six patients with four potentially pathogenic *RECQ1* mutations: one frameshift deletion (c.974_977delAAGA), two splicing site mutations (c.394+1G>A, c.867+1G>T) and one nonsense mutation (c.796C>T, p.Q266*). Two mutations (c.867+1G>T and p.Q266*) were seen in more than one patient. They also found 14 missense mutations, two in the RQC domain [65]. In 2017, Sun et al. again screened 62 known cancer-susceptibility genes among 8085 unselected Chinese patients with breast cancer, and they found 285 *BRCA2*, 146 *BRCA1*, 56 *PALB2*, 38 *TP53,* and 30 *RECQ1* mutated patients. *RECQ1* had 15 different pathogenic mutations: six nonsense mutations, three frameshift insertions, one splicing mutation, and five deleterious missense mutations [66]. In 2018, Tervasmaki et al. reported a *RECQ1* missense mutation (p.I156M) among 6 of 1946 Finnish patients with breast cancer, whereas none of the 1408 controls carried that mutation [67].

These studies from five populations of different ancestry suggested that *RECQ1* is a breast cancer susceptibility gene. However, a few studies have presented conflicting evidence. In 2017, Bogdanova et al. have examined the frequency of the recurrent Polish *RECQ1* mutation, c.1667_1667+3delAGTA, in Belarusian and German breast cancer patients, and found the mutation in 9 of 2596 (0.35%) cases and 6 of 2132 (0.28%) controls, with an adjusted OR = 1.23, *p* = 0.69 [68]. Recently, Li et al., in 2018, screened 4536 Australian women who had a previous negative result from *BRCA1* and *BRCA2* mutation testing and a family history of breast cancer and a personal history of breast cancer (>95%) or ovarian cancer. They also examined 4576 cancer-free female controls. They found 13 loss-of-function (7 nonsense, 1 frameshift, and 5 essential splice-site mutations) *RECQ1* mutations in the cases (0.29%), compared to 25 loss-of-function (18 nonsense, 4 frameshift, and 3 essential splice-site mutations) mutations in the controls (0.55%) with an adjusted OR = 0.52, *p* = 0.072. They did not find any carriers of the Quebec founder mutation (c.643C>T) in either the cases or controls and only 2 cases and 1 control were found with the Polish founder mutation (c.1667_1667+3delAGTA). Based on these findings, Li et al. [69] concluded that *RECQ1* is not associated with breast cancer risk in Australia. Notably, in the Li et al. study, a truncating mutation (c.1859C>G, p.S620*) was the most common mutation in both cases (6/13) and controls (16/25) [69]. This mutation is seen at a frequency of 0.20% in the Exome Aggregation Consortium (ExAC) European dataset [70]. Nguyen-Dumont et al., in 2018, screened 338 breast cancer patients and 89 ovarian cancer patients from southwestern Poland and west Ukraine, and they did not identify any carriers with *RECQ1* mutation c.1667_1667+3delAGTA [71]. Hilz et al., in 2019, also examined 715 breast cancer patients and 916 controls from Latvia, and they did not find any carriers with *RECQ1* mutation c.1667_1667+3delAGTA in their case group, but they found 2 in their control group [72]. Table 2 summarizes all the reported studies.

Collectively, these studies place *RECQ1* among the list of genes responsible for moderate risk susceptibility to breast cancer, which accounts for up to 5% of the inherited familial risk [73]. Case-controlled studies in large and ethnically diverse populations will identify additional rare variants of *RECQ1* and further confirm the risk association between *RECQ1* mutations and breast cancer.

Although *RECQ1* germline mutations are rare, they are likely to be clinically relevant, given the functional importance of RECQ1 in genome maintenance mechanisms. The pathogenic *RECQ1* missense mutations (A195S, R215Q, R455C, M458K, and T562I) identified in breast cancer patients include highly conserved amino acid residues in catalytic domains of RECQ1 (Figure 1B), and the recombinant proteins show complete (R215Q, R455C, M458K, and T562I) or partial (A195S) loss of helicase activity in vitro. By using the CRISPR/Cas9 system to completely ablate RECQ1 expression, and reconstitution with wild-type RECQ1 or breast cancer risk-associated RECQ1 variants, we found that MDA-MB-231 breast cancer cells expressing these missense RECQ1 mutants are more sensitive to gemcitabine, a drug that induces replication-associated DNA damage and is used for triple-negative breast cancer [34]. This indicates that the helicase activity of RECQ1 may be essential in resolving DNA damage and cancer cell survival. These initial studies suggest that missense mutations in RECQ1 impair critical DNA repair functions, thereby leading to breast cancer susceptibility. However, the functional consequences associated with other missense variants and truncating variants of RECQ1 are still unclear.

## 7. Outlook for RECQ1 Relationship with Cancer

RECQ1 maintains genome integrity by governing diverse cellular functions ranging from DNA replication, repair of endogenously and exogenously induced DNA damage, regulation of cell cycle and growth, and telomere stability to gene transcriptional regulation. Consistent with its putative tumor suppressor role, germline mutations in *RECQ1* have been identified in hereditary breast cancer patients. Although several reports have now suggested *RECQ1* as a breast cancer susceptibility gene, a few studies have reported conflicting evidence. In this regard, further studies are required to better establish the functional impact of mutations in *RECQ1* in breast and other cancer types. It will be insightful to investigate the risk of other malignancies within families carrying breast cancer risk-associated variants of *RECQ1*. This is especially important since genetic susceptibility in breast cancer is shared with other tumor types [73,74]. However, the association of *RECQ1* with other cancer types can be difficult to assess, especially if it turns out to be a rare risk gene with moderate penetrance. It will also be important to investigate how a given *RECQ1* variant impacts breast cancer risk in combination with other genetic risk factors, lifestyle factors, and family history in terms of absolute risk. Genetic alterations that drive the malignant transformation of a normal cell and the process of tumorigenesis comprise both germline (inherited) and somatic (acquired) mutations [75]. While the clinical significance of germline mutations of the *RECQ1* gene is now unfolding, whether harboring somatic *RECQ1* mutations has the same biological effects as their germline counterparts remain to be determined. Identifying genetic modifiers that potentiate the effect of the RECQ1 variant in patients or attenuate the effects of the RECQ1 risk allele in unaffected mutation carriers in controls may provide the best opportunity to convert insights from rare variants into discoveries of clinical and biological significance. Functional follow-up analyses of this genetic discovery through novel experimental models, such as CRISPR/Cas9 mediated genome editing, will be instrumental in addressing some of these questions. In addition to mutations, altered RECQ1 expression may be correlated with therapeutic response and disease prognosis in sporadic breast and other cancers. Determining the regulators of RECQ1 expression and how they are co-opted during tumorigenesis could lead to novel therapeutic strategies. Given the role of RECQ1 in DSB repair, it will be useful to explore its use in cancer therapeutics. It will be important to determine the biological consequences arising from the loss of RECQ1 interaction with other proteins. Comprehensive analysis of the expression and mutation of RECQ1 and its interaction networks is required to elucidate the underlying molecular mechanisms of RECQ1 functions in genome maintenance and tumor suppression.

## Figures and Tables

**Table 1:** Alphabetical list of the known interacting partners of RECQ1.

Protein	Functional Interaction	Reference
BAF	Unknown	[24]
DDB1	Unknown	[25]
EXO-1	RECQ1 stimulates EXO-1 nuclease activity	[26]
FEN-1	RECQ1 stimulates FEN-1 nuclease activity	[27]
Importin-α (Qip1 and Rch1)	Unknown	[23]
Ku70/80	RECQ1 regulates DNA binding of Ku70/80	[28]
MLH1-PMS2	Unknown	[26]
MSH2/6	MSH2/6 stimulates RECQ1 helicase activity	[26]
PARP-1	PARP-1 regulates ATPase and branch migration activities of RECQ1	[19]
	RECQ1 regulates poly-ADP-ribosylation activity of PARP-1	[29]
RAD51	Unknown	[18]
RPA	RPA stimulates RECQ1 helicase activity	[30]
Top3α	Unknown	[31]

**Table 2:** Reported breast cancer risk-associated *RECQ1* mutations.

Study	Mutation	Change in Protein	No. of Carriers/Total No. of Cases	No. of Carriers/Total No. of Controls
Cybulski et al. (2015) [5]	c.643C>T c.1667_1667+3delAGTA	p.R215 * p.K555delinsMYKLIHYSFR	7/1013 30/13136	1/7136 2/4702
Sun et al. (2015) [6]	c.383T>G	p.L128 *	1/448	0/1588
	c.516G>A	p.W172 *	1/448	0/1588
	c.796C>T	p.Q266 *	1/448	0/1588
	c.395-2A>G	p.G132fs *	1/448	0/1588
	c.644G>A	p.R215Q	1/448	0/1588
	c.1363C>T	p.R455C	1/448	1/1588
	c.1373T>A	p.M458K	1/448	0/1588
	c.1685C>T	p.T562I	1/448	0/1588
	c.583G>T	p.A195S	1/448	0/1588
Kong et al. (2016) [65]	c.974_977delAAGA	p.K325Tfs *	1/1110	0/88
	c.394+1G>A	-	1/1110	0/88
	c.867+1G>T	-	2/1110	0/88
	c.796C>T	p.Q266 *	2/1110	0/88
Sun et al. (2017) [66]	c.1856dupA	p.N619fs	1/8085	0/0
	c.C1685T	p.T562I	1/8085	0/0
	c.G1398A	p.W466 *	2/8085	0/0
	c.T1373A	p.M458K	1/8085	0/0
	c.C1363T	p.R455C	1/8085	0/0
	c.C796T	p.Q266 *	3/8085	0/0
	c.G644A	p.R215Q	10/8085	0/0
	c.G583T	p.A195S	1/8085	0/0
	c.A577T	p.K193 *	1/8085	0/0
	c.G516A	p.W172 *	2/8085	0/0
	c.490_491insAAATGCTT	p.S164_S165delins *	1/8085	0/0
	c.395-2A>G	-	2/8085	0/0
	c.T383G	p.L128 *	1/8085	0/0
	c.189_190insATGATTCT	p.S64fs	1/8085	0/0
	c.120dupA	p.V41fs	2/8085	0/0
Tervasmaki et al. (2018) [67]	c.468T>G	p.I156M	6/1946	0/1408
Bogdanova et al. (2017) [68]	c.1667_1667+3delAGTA	p.K555delinsMYKLIHYSFR	9/2596	6/2132
Li et al. (2018) [69]	c.85dupA	p.T29Nfs *	0/4536	1/4576
	c.426delT	p.M144Wfs *	0/4536	2/4576
	c.501+1G>A	-	1/4536	0/4576
	c.701-2A>C	-	1/4536	0/4576
	c.730C>T	p.Q244 *	0/4536	1/4576
	c.796C>T	p.Q266 *	0/4536	1/4576
	c.962_965delGTTT	p.C321Ffs *	1/4536	0/4576
	c.1098+1G>A	-	0/4536	1/4576
	c.1355+1G>A	-	1/4536	1/4576
	c.1476C>G	pY492 *	1/4536	0/4576
	c.1667_1667+3delAGTA	p.K555delinsMYKLIHYSFR	2/4536	1/4576
	c.1841_1844dupAGAT	p.M615Ifs *	0/4536	1/4576
	c.1859C>G	p.S620 *	6/4536	16/4576
Nguyen-Dumont et al. (2018) [71]	c.1667_1667+3delAGTA	p.K555delinsMYKLIHYSFR	0/427	0/0
Hilz et al. (2019) [72]	c.1667_1667+3delAGTA	p.K555delinsMYKLIHYSFR	0/715	2/916

*—termination; del—deletion; ins—insertion; fs—frameshift; dup—duplication. A silent mutation contributing to no change in protein is indicated by -.
